# Potential of cold plasma to control *Callosobruchus chinensis* (Chrysomelidae: Bruchinae) in chickpea cultivars during four year storage

**DOI:** 10.1038/s41598-021-92792-x

**Published:** 2021-06-28

**Authors:** F. L. Pathan, R. R. Deshmukh, U. S. Annapure

**Affiliations:** 1 Department of Food Engineering and Technology, Institute of Chemical Technology, N.P Marg, Matunga, Mumbai, 400019 India; 2 Department of Physics, Institute of Chemical Technology, N.P Marg, Matunga, Mumbai, 400019 India

**Keywords:** Biotechnology, Zoology, Physics

## Abstract

Cold plasma has proven itself as a promising method of food preservation by controlling food spoilage bacteria at very low temperatures. It is showing potential for insect control. Synthetic pesticides are mostly used to control *Callosobruchus chinensis L. *(Chrysomelidae: Coleoptera) to which it has developed resistance*.* The prospective potential of cold plasma treatment to control pulse beetle infestation of chickpea in the storage for about four years of plasma treatment was studied. The four chickpea cultivars were treated with cold plasma at different power 40, 50, and 60 W each for 10, 15, 20 min. Plasma treated and untreated chickpeas were stored in an airtight ziplock pouch. At regular intervals, the grains were observed for infestation. It was found most effective in controlling the pulse beetle infestation of treated chickpea samples. While plasma untreated chickpeas were attacked and damaged mostly by pulse beetle within the first quarter of the storage study. To avoid the problems created by the use of pesticides cold plasma treatment is found to be the best alternative in the protection of chickpea invasion by pulse beetle during a longer storage period. The findings in the present research may be used for the preparation of legumes which may also soak and cook faster like quick-cooking legumes and preserved for years without invasion of pulse beetle.

## Introduction

Through symbiotic nitrogen fixation, legumes derive their nitrogen requirement. They contribute annually 50–70 million tonnes of nitrogen to the agricultural ecosystem^1^. The world grows and consumes chickpea (*Cicer arientinum* L.). India is a major chickpea-producing country. Its chickpea production is 9.9 million tons with 10.041 t/ha productivity from 9.5 million ha area^2^. There is an extensive presence of stored product insects in milling, processing, and warehouse facilities^3^. During storage of chickpea seeds fungal contamination and *callosobruchus* infestation cause quantitative as well as qualitative losses^4^. The bruchid *Callosobruchus* spp. are the main storage insect pest of chickpea. Infestation in grain legumes commences in the field even before the crop harvest, and then they multiply quite fast in storage, resulting in heavy weight loss, lower germination potential, and affect its quality^5^. Banga et al. (2019) found that acoustic detection method may provide rapid and non-destructive infestation detection in bulk stored legumes^6^. In legume seeds the Seed germination decreases with the degree of infestation^7^.

Insect species causes post-harvest losses of the food grains in the field as well as in storage created challenges since time immemorial. Insecticide misuse has led to increased levels of resistance to insecticides, emergence of secondary pests, loss of biodiversity, and a rise in human health hazards^8,9^. Honey bees are boon for the biodiversity and economy through their pollination services in forests and crops. However, the use of pesticides has adversely affected queen production^10^. Which has the potential to disturb the honey bee ecosystem with the potential to disturb all other ecosystems including human beings. People are increasingly desirous to get food with low or no pesticide residues^8^. Reducing the pest with increasing natural enemies will modify the environment through conservation biological control^11^. Natural pest control can be increased to reduce dependence on pesticides for insect control^12^.

Nowadays to avoid and reduce the problems created by synthetic pesticides the use of sex pheromones, genome editing technique, use of nanocarrier to introduce peptide which will interfere with the immunity and reproduction of insects, selection of suitable target genes based insect control strategy- RNA interface, Sterile Insect Technique, nanoencapsulation of botanical compounds, use bio fumigants, essential oils has been studied and tried at depth for the management of various insect pests^9,13–18^.

Non-thermal plasma or cold plasma works at ambient temperature conditions. It generates energetic electrons that collide with gas molecules. This results in dissociation, excitation, and ionization of gas molecule^19^. Initially after suing plasma applications to medicine and biology^20^; agricultural applications^21^; it is used for insect control^22^. Through reactive oxygen stress in the Indian meal moth *(Plodia interpunctella*) larvae, it causes oxidative damage^23^. Insect pest and pathogens affect agricultural production and the entire food industry. Synthetic fungicides are the only promising solution for the same till today. The cold plasma is a novel suitable solution for it^24^. Cold plasma is used for conditioning and microbiological decontamination of biomaterials including food^25^. Sarangpani et al., (2017) studied the degradation efficacy of pesticides in blueberries after 80 kV and 5 min in an in-package high voltage dielectric barrier discharge plasma reactor^26^. Plasma treatment was found to be effective in improving the germination efficiency of seeds^27^. Plasma-treated tap water used for the control of mealybug (*Planococcus citri*) infestation in Nerium (*Nerium oleander)*^28^. It is also used as an advanced oxidation process to degrade the pesticides^29^. Plasma generation device, plasma exposure time, plasma power, and the carrier gas composition, influence the type and concentration of reactive species (e.g. ROS and RNS) and the overall efficiency of cold plasma degradation for a specific pesticide or allergen^30^. In rice, wheat, corn, barley and oats cold plasma treatment ably reduced fungi, bacteria, and their spores on grain surfaces. It detoxify mycotoxins and control insect pests^31^. The cold plasma improves seed germination, the yield of agricultural products food quality, and nutritional security by the use of surface sterilization and inclusion of active nutrients in the plant system. It does not create any environmental or health hazard issue^32^. Plasma treatment used in wheat to control confused flour beetle (*Tribolium confusum*) and Mediterranean flour moth (*Ephestia kuehniella*)^33^. The eggs, grub and adult stages of red flour beetle (*Tribolium castaneum*, Coleoptera: Tenebrionidae) by non-thermal plasma^34^.

Cold plasma processing has attracted many researchers nowadays through its effective outcomes in the field of food processing especially for storage insect or pests control. The objectives of the present investigation were to evaluate the *C. chinensis* effects during the storage period of cold plasma treated four chickpea cultivars. The outcomes of this research will be helpful to preserve the chickpea for about more than four years and save the losses of chickpea due to *C. chinensis.*

## Results

### Quarterly assessment of grain damage percentage

There were no significant difference in the control samples for percent grain damage of all cultivars (Table 1.). (Kripa: d.f. = 2, 6; F = 0.0; *p* = 1.0, Virat: d.f. = 2, 6; F = 0.0; *p* = 1.0, Vishal: d.f. = 2, 6; F = 0.0; *p* = 1.0, Rajas: d.f. = 2, 6 F = 0.0; *p* = 1.0). There were no significant difference in the treated samples for percent grain damage of all cultivars (Table 1).

**Table 1:** Comparison between plasma untreated and plasma-treated samples of six chickpea cultivar during 4-year storage for grain damage percentage, number of eggs/grain. Number of holes/ grain and percent grain weight loss.

Plasma treatment		Grain damage (%)	Number of eggs/grain	Number of holes/grain	Grain weight loss (%)
Exposure Power (W)	Exposure Time (min)				
**Kripa**					
Control	–	100 ± 0.81^a^	25.26 ± 0.11^a^	3.52 ± 0.03^a^	62.99 ± 1.42^b^
40	10	0	0	0	6.4 ± 0.23^a^
	15	0	0	0	6.43 ± 0.26^a^
	20	0	0	0	6.55 ± 0.36^a^
50	10	0	0	0	4.39 ± 0.16^a^
	15	0	0	0	6.77 ± 0.19^a^
	20	0	0	0	6.79 ± 0.19^a^
60	10	0	0	0	6.54 ± 0.12^a^
	15	0	0	0	6.57 ± 0.19^a^
	20	0	0	0	6.59 ± 0.14^a^
**Virat**					
Control	*-*	98 ± 0.81^a^	24.23 ± 0.05^a^	3.14 ± 0.02^a^	62.66 ± 1.42^b^
40	10	0	0	0	9.03 ± 0.57^a^
	15	0	0	0	9.65 ± 0.83^a^
	20	0	0	0	10.52 ± 0.81^a^
50	10	0	0	0	9.26 ± 0.28^a^
	15	0	0	0	9.92 ± 0.95^a^
	20	0	0	0	10.55 ± 0.94^a^
60	10	0	0	0	9.49 ± 0.44^a^
	15	0	0	0	10.49 ± 0.65^a^
	20	0	0	0	12.34 ± 0.86^a^
**Vishal**					
Control	-	95 ± 0.81^a^	24.6 ± 0.02^a^	2.9 ± 1.33^b^	62.87 ± 1.42^b^
40	10	0	0	0	8.86 ± 0.12^a^
	15	1	4.28 ± 1.33^a^	0.01 ± 0.00	8.11 ± 0.74^a^
	20	2	5.12 ± 1.33^a^	0.02 ± 0.00	8.10 ± 0.72^a^
50	10	0	0	0	8.04 ± 0.58^a^
	15	0	0	0	7.15 ± 0.13^a^
	20	0	0	0	8.00 ± 0.59^a^
60	10	0	0	0	8.02 ± 0.47^a^
	15	0	0	0	7.54 ± 0.33^a^
	20	0	0	0	7.56 ± 0.30^a^
Plasma treatment		Grain damage (%)	Number of eggs/grain	Number of holes/grain	Grain weight loss (%)
Exposure Power (W)	Exposure Time (min)				
**Rajas**					
Control	–	97 ± 0.81^a^	25.44 ± 0.02^a^	3.12 ± 0.05^a^	62.87 ± 0.69^b^
40	10	0	0	0	11.30 ± 0.69^a^
	15	0	0	0	12.46 ± .17^a^
	20	0	0	0	12.30 ± 0.91^a^
50	10	0	0	0	13.71 ± 0.87^a^
	15	0	0	0	12.49 ± 0.37^b^
	20	0	0	0	13.13 ± 0.58^a^
60	10	0	0	0	14.83 ± 0.71^a^
	15	0	0	0	15.01 ± 0.14^a^
	20	0	0	0	13.46 ± 0.17^a^

All the data are expressed as mean ± standard error. Means with the different superscript letters in a column differ significantly (*P* < 0.05).

### Quarterly assessment of grain weight loss percentage

There were significant difference in the control samples for grain weight loss of all cultivars except and Rajas (Table 1.) (Kripa: d.f. = 2, 6; F = 0.66; *p* = 0.045 , Virat: d.f. = 2, 6; F = 0.13; *p* = , Vishal: d.f. = 2, 6; F = 0.107; *p* = 0.048, Rajas: d.f. = 2, 6; F = 0.559; *p* = 0.170 ). There were no significant difference in the treated samples for grain weight loss of all cultivars (Table 1.) (Kripa: d.f. = 2, 6; F = 1.842; *p* = 0.045 , Virat: d.f. = 2, 6; F = 4.109; *p* = , Vishal: d.f. = 2, 6; F = 0.574; *p* = 0.048, Rajas: d.f. = 2, 6; F = 6.798; *p* = 0.170 ).

There were no significant difference in the samples for grain weight loss in all the treatment times when treated at 40 W of cold plasma to Kripa except 40 W 15 min treatment (Table 1.) (Kripa control: d.f. = 2, 6; F = 0.272; *p* = 0.771, Kripa 40 W, 10 min.: d.f. = 2, 6; F = 9.387; *p* = 0.014, Kripa 40 W, 15 min. d.f. = 2, 6; F = 8.872; *p* = 0.016, Kripa 40 W, 20 min. d.f. = 2, 6; F = 0.356; *p* = 0.714). There were no significant difference in the samples for grain weight loss in all the treatment times when treated at 50 W cold plasma (Table 1.) (Kripa control: d.f. = 2, 6; F = 0.272; *p* = 0.771, Kripa 50 W, 10 min.: d.f. = 2, 6; F = 0.797; *p* = 0.493, Kripa 50 W, 15 min. d.f. = 2, 6; F = 1.096; *p* = 0.393, Kripa 50 W, 20 min. d.f. = 2, 6; F = 1.190; *p* = 0.367). There were no significant difference in the samples for grain weight loss in all the treatment times when treated at 60 W cold plasma (Table 1.) except 60 W 15 min. (Kripa control: d.f. = 2, 6; F = 0.272; *p* = 0.771, Kripa 60 W, 10 min.: d.f. = 2, 6; F = 1.250; *p* = 0.352, Kripa 60 W, 15 min. d.f. = 2, 6; F = 5.170; *p* = 0.050, Kripa 60 W, 20 min. d.f. = 2, 6; F = 0.089; *p* = 0.916).

There were no significant difference in the samples for grain weight loss in all the treatment times when treated to Virat at 40 W cold plasma treatment except for 10 min. Where the grain loss is lowest at 10 min treatment and was highest at 20 min treatment (Table 1.). (Virat control: d.f. = 2, 6; F = 0.318; *p* = 0.739, Virat 40 W, 10 min.: d.f. = 2, 6; F = 6.221; *p* = 0.034, Virat 40 W, 15 min. d.f. = 2, 6; F = 2.215; *p* = 0.190, Virat 40 W, 20 min. d.f. = 2, 6; F = 2.616; *p* = 0.152). There were no significant difference in the samples for grain weight loss in all the treatment times when treated at 50 W cold plasma (Table 1.) (Virat control: d.f. = 2, 6; F = 0.318; *p* = 0.739, Virat 50 W, 10 min.: d.f. = 2, 6; F = 0.196; *p* = 0.827, Virat 50 W, 15 min. d.f. = 2, 6; F = 0.245; *p* = 0.790, Virat 50 W, 20 min. d.f. = 2, 6; F = 1.204; *p* = 0.364). There were no significant difference in the samples for grain weight loss in all the treatment times when treated at 60 W cold plasma (Table 1.) (Virat control: d.f. = 2, 6; F = 0.318; *p* = 0.739, Virat 60 W, 10 min.: d.f. = 2, 6; F = 1.703; *p* = 0.260, Virat 60 W, 15 min. d.f. = 2, 6; F = 1.708; *p* = 0.259, Virat 60 W, 20 min. d.f. = 2, 6; F = 0.111; *p* = 0.897).

There were no significant difference in the samples for grain weight loss in all the treatment times when treated at 40 W cold plasma to Vishal (Table 1.) (Vishal control: d.f. = 2, 6; F = 0.318; *p* = 0.739, Vishal 40 W, 10 min.: d.f. = 2, 6; F = 0.497; *p* = 0.631, Vishal 40 W, 15 min. d.f. = 2, 6; F = 1.025; *p* = 0.414, Vishal 40 W, 20 min. d.f. = 2, 6; F = 0.413; *p* = 0.679). There were no significant difference in the samples for grain weight loss in all the treatment times when treated at 50 W cold plasma (Table 1.) (Vishal control: d.f. = 2, 6; F = 0.318; *p* = 0.739, Vishal 50 W, 10 min.: d.f. = 2, 6; F = 0.612; *p* = 0.573, Vishal 50 W, 15 min. d.f. = 2, 6; F = 0.893; *p* = 0.458, Vishal 50 W, 20 min. d.f. = 2, 6; F = 0.119; *p* = 0.890). There were no significant difference in the samples for grain weight loss in all the treatment times when treated at 60 W cold plasma (Table 1.) (Vishal control: d.f. = 2, 6; F = 0.318; *p* = 0.739, Vishal 60 W, 10 min.: d.f. = 2, 6; F = 2.900; *p* = 0.131, Vishal 60 W, 15 min. d.f. = 2, 6; F = 0.515; *p* = 0.622, Vishal 60 W, 20 min. d.f. = 2, 6; F = 0.729; *p* = 0.521).

There were no significant difference in the samples for grain weight loss in all the treatment times when treated to Rajas at 40 W cold plasma (Table 1.) (Rajas control: d.f. = 2, 6; F = 0.713; *p* = 0.527, Rajas 40 W, 10 min.: d.f. = 2, 6; F = 4.288; *p* = 0.070, Rajas 40 W, 15 min. d.f. = 2, 6; F = 3.367; *p* = 0.105, Rajas 40 W, 20 min. d.f. = 2, 6; F = 0.706; *p* = 0.090). There were no significant difference in the samples for grain weight loss in all the treatment times when treated at 50 W cold plasma treatment except for 15 min. (Table 1.) (Rajas control: d.f. = 2, 6; F = 0.713; *p* = 0.527, Rajas 50 W, 10 min.: d.f. = 2, 6; F = 0.181; *p* = 0.839, Rajas 50 W, 15 min. d.f. = 2, 6; F = 13.02; *p* = 0.007, Rajas 50 W, 20 min. d.f. = 2, 6; F = 2.865; *p* = 0.134). There were no significant difference in the samples for grain weight loss in all the treatment times when treated at 60 W cold plasma treatment. (Table 1.) (Rajas control: d.f. = 2, 6 F = F = 0.713; *p* = 0.527, Rajas 60 W, 10 min.: d.f. = 2, 6; F = 1.914; *p* = 0.228, Rajas 60 W, 15 min. d.f. = 2, 6; F = 3.828; *p* = 0.085, Rajas 60 W, 20 min. d.f. = 2, 6; F = 2.937; *p* = 0.129).

### Quarterly assessment of the number of eggs/grain

There were no significant differences in the control samples for number of eggs per grain of all cultivars (Table 1.). Eggs laid in all the plasma treated samples for all the four varieties were 0 except it was 4.28 to 5.12 in variety Vishal for 40 W 15 min and 40 W 20 min treatment. (Kripa: d.f. = 2, 6; F = 1.666; *p* = 0.266 , Virat: d.f. = 2, 6; F = 0.094; *p* = 0.912, Vishal: d.f. = 2, 6; F = 0.527; *p* = 0.615, Rajas: d.f. = 2, 6; F = 0.843; *p* = 0.476). There were no significant difference in the treated samples for number of eggs per grain of all cultivars (Table 1).

### Quarterly assessment of the number of holes/grain

There were no significant differences in the control samples for number of holes per grain of all cultivars except Vishal (Table 1.) (Kripa: d.f. = 2, 6; F = 0.613; *p* = 0.572 , Virat: d.f. = 2, 6; F = 4.742; *p* = 0.058 , Vishal: d.f. = 2, 6; F = 17.106; *p* = 0.003, Rajas: d.f. = 2, 6; F = 1.705; *p* = 0.259). There were no significant difference in the treated samples for number of holes per grain of all cultivars (Table 1.)

There were no significant differences in the samples for number of holes per grain in all the treatment times when treated Vishal at 40 W cold plasma (Table 1.) (Vishal control: d.f. = 2, 6; F = 0.542; *p* = 0.608, Vishal 40 W, 20 min. d.f. = 2, 6; F = 0.214; *p* = 0.813). There was no significant difference in the samples for the number of holes per grain in all the treatment times when treated at 50 W cold plasma. There was no significant difference in the samples for the number of holes per grain in all the treatment times when treated at 60 W cold plasma. (Table 1).

## Discussion

The plasma treatment was found significantly effective over control with regards to check the grain damage in most of the chickpea samples (Table 1, Supplementary Table S1, and Supplementary Figure S1). Grain damage percentage in plasma untreated grains was much higher in the first quarter of the assessment. It was 100% in Kripa, 99% in Virat, 97% in Rajas, 95% in Vishal. While in plasma-treated grains except in Vishal (40 W, 15, and 20 min) grain damage was from 1 to 3%. While in rest all treated samples of all the cultivars the grain damage percent was found to be 0% in the entire storage period of the 48 months in all the treated grains after treatment with 40–60 Watts for10–20 min.

As compared to the plasma this system can be applied for pest control. But only if the seeds before sowing are treated with plasma can make those self-stable to fight with on-farm or in storage diseases and pests. Moreover, it also improves a wide range of qualities of seeds. Natural pest optimization can be obtained through establishing non-crop areas, temporal crop rotation control, and low-impact tillage, at local and landscape scales. They suggested that multiple enemies attack pests during different periods of their occurrence in the field which is responsible to improve biological control efficacy^12^.

Though it looks simple to suggest as above, the practices mentioned to maintain the landscape require discipline and unity in the farmers and to follow all the practices unanimously. When cold plasma can be used for pest control from sowing the seeds to harvested seeds. This will be the completion of one cycle of cold plasma use for pest and disease control. Many such cycles needed to make it and habit for generations of farmers. This will surely make pests disappear from landscapes forever and the use of pesticide and our dependence on it can be reduced magnificently. The process efficacy for a wide range of plasma applications, like antimicrobial, pesticidal, food functionalization, and waste treatment has been demonstrated through multidisciplinary scientific efforts^26^. The chitosan nanoparticles functionalized with β-cyclodextrin prepared which has carvacrol and linalool. The toxicity and biological activity were evaluated. The toxicity decreased when the compounds were nano-encapsulated^9^. No such problem of the requirement of encapsulation is required when cold plasma is used to treat the seeds to control the pest and diseases and subsequent reduction in toxicity to the pest.

Results in our investigation for all the parameters viz. grain damage percentage, grain weight loss percentage, number of eggs/grain, and number of holes/grain are in close proximity with results obtained by Rahman et al.^34^. Only in cases where the number of holes/grain and the number of eggs per grain occurs in plasma treated samples, it may be due to the infestation of those samples before treatment, but non-occurrence of further damage of it shows the 100% control of plasma treatment over pulse beetle^34^.

Grain weight loss percentage in plasma untreated grains was much higher in the first quarter of assessment and thereafter it was occurred decreasing slowly throughout the storage period. It was 56.85–62.87% in Rajas, 56.83–62.86% in Vishal, 56.87–62.99% in Kripa, 58.14–62.64% in Virat in control samples. While in plasma-treated grains the weight loss percentage was found decreasing steadily as the bruising has been occurred to the grains due to plasma treatment in the entire storage period of 48 months. In Kripa it is decreased from 6.4 -6.79%, in Vishal 7.15 -9.72%, in Virat 9.05 -10.53%, in Rajas 11.32 -15.00% after treatment with 40–60 Watts for 10–20 min (Table 1, Supplementary Table S2 and Supplementary Figure S2).

The Trojan female technique a mutation in the mitochondrial cytochrome b gene (mt: Cyt-b) was studied^7^. It reduces male fertility. They further mentioned that the effects of this mutation were moderated by the nuclear background and thermal environment. The fertility of males carrying the mutation was invariably reduced relative to controls^7^.

It does not seem practically feasible to carry out mutation, peptide introduction, or genome editing (Guo et al., 2015, Czarniewska et al., 2019, Koutroumpa et al., 2016) to control insects that act as a pest on-farm and in or during storage Rather the technology like cold plasma can be invariably used for control of all the insects acting as a pest. Results in our investigation for all the parameters viz. grain damage percentage, grain weight loss percentage, number of eggs/grain, and number of holes/grain are near the results obtained by Rahman et al. (2018)^34^.

Loganathan et al. (2011)^37^ used thermal disinfestation for insect control. They held eggs, larvae, pupae, and adults at 42 °C or 0 °C for varying durations. They found that pupae and adults were equally heat tolerant. According to them LT _50_ at 42 °C for eggs, larvae, pupae, and adults were 18, 57, 78, and 71 h, respectively. The LT _50_ at 0 °C for eggs, larvae, pupae, and adults were 3, 8, 10, and 4 d, respectively. Pupa was the most cold-tolerant stage. Comparing with thermal processing of insects plasma processing was found to be more effective as it is done at lower temperature and for less time^37^. *Datura alba* leaf extracts at 2.5% shown 33.5 and 45% mortality in *T. granarium* and *S. oryzae* after 7 days of exposure, respectively^38^. Spinosad controlled *C. maculatus* throughout the 6 months of cowpea storage whereas deltamethrin failed to control *C. maculatus* after 3 months of storage^39^. Ozone at higher concentrations like 47–106 ppm could damage equipment in only 2 months by corrosion. Therefore Jian et al.^40^ recommended ton use it ≤ 50 ppm for control of stored grain pests in the stored grain industry.

In plasma, untreated grains number of eggs/grain was much higher in the first quarter of assessment. It was 2.90% in Vishal, 3.12% in Rajas, 3.14% in Virat, 3.51% in Kripa. In plasma-treated grains the number of eggs/grain was found to be in Vishal ranging from 4.28 to 5.12%, which might be laid before the plasma treatment, hence it remains constant on the grain without fertilization of eggs for the entire storage period in grains after plasma treatment with 40–60 Watts for 10–20 min. While in plasma-treated samples remaining grains of all the cultivars the number of eggs/grain was found to be 0% in the entire storage period of the 48 months in plasma-treated grains with 40–60 Watts for10–20 min (Table 1, Supplementary Table S3 and Supplementary Figure S3).

The LC_50_ and LC_*95*_ of the essential oil of *V. arborea* and *α-bisabolol* were 5.23 and 12.97 μL L^−1^ of air and 2.47 and 8.82 μL L^−1^ of air, respectively. Thus the Increased concentrations of the essential oil reduced the instantaneous rate of population growth, rate of development, oviposition, and the number of eggs of *C. maculatus*, and therefore they have the potential for pest control^18^. Seventy volatiles were identified from which the six putative attractants and 6 repellents were emerged out, useful biocontrol tools^41^.

Many plants cannot yield the essential oil more than 1% in this condition how to extract essential oil commercially because quintal of biomass can give only less than 1 kg essential oil. These all essential oils are very volatile in nature and hence these are also known as volatile oils. The process to produce these are costlier. Therefore when it has to be produced and used commercially, it will be available at very higher costs. How it is then accepted by farmers? Simply if in the coming days the seed manufacturing companies can treating their seeds with cold plasma treatment which will be able to control diseases and pests during storage and on the farm as a crop. Agricultural expansion at the cost of natural habitats is the era of human-dominated landscapes^42^. This domination which had created many problems by impacting the environment and lives of ourselves can be reversed if we can get greater outputs from cold plasma and we would able soon to use it commercially as the method of pest control.

The results in this experiment showing no egg /grain on plasma-treated chickpea are in confirmation with Shahrzad et al. (2015) who treated instar larvae among wheat grains. They further reported that the mortality reached 100% after 20 s for both the pests called confused flour beetle and Mediterranean flour moth^33^. Rahman et al. (2018) investigated the effect and mechanisms of the low-pressure dielectric barrier where egg, larval, and adult stages of red flour beetle were treated by non-thermal plasma. In all flour beetles' stages, 100% mortality was achieved depending on the plasma exposure time and plasma intensity. Moreover, they stated that an optimal effect of plasma with an impact on all stages of red flour beetle (*T. castaneum)* was observed^34^. Results in our investigation for all the parameters viz. grain damage percentage, grain weight loss percentage, number of eggs/grain, and number of holes/grain are in close proximity with results obtained by Rahman et al. (2018).

In plasma, untreated grains number of holes/grain was much higher in the first quarter of assessment. It was 24.23 in Virat, 24.6 in Vishal, 25.26 in Kripa, and 25.44 in Rajas. In plasma-treated grains the number of holes/grain was found to be very less in Vishal ranging from 0.01 to 0.02, which might be bored by pulse beetle or bruchid before the plasma treatment, hence it remains constant on the grain for the entire storage period after its plasma treatment with 40–60 Watts for 10–20 min. While in plasma-treated remaining grains of all the cultivars the number of holes/grain was found to be 0 in the entire storage period of the 48 months in grains after plasma treatment with 40–60 Watts for 10–20 min (Table 1, Supplementary Table S4 and Supplementary Figure S4).

The larvae which hatch from the eggs of the *Callosobruchus* species needed to penetrate in the seed to survive^43^. Most of the plasma-treated samples where no holes found on grain shows that in it the eggs are not hatched into larvae. The plasma-treated samples where holes are found in grain are so negligible, it indicates it might be possibly due to infestation of these samples before plasma treatment up to the larval stage, but it gets eradicated due to plasma treatment and no occurrence of subsequent life stages of pulse beetle exhibited. Mahendran et al. (2016) used, electrode gap (3 to 5 cm), exposure time (1 to 5 min), and applied voltage (1000 to 2500 V) while studying mortality of red flour beetle (*Tribolium castaneum*). They reported that there is a significant increase in the mortality rate of an adult with an increase in applied voltage, exposure time, and decrease in electrode distance^22^. The powders of *C. camphora*, *O. basilicum*, and *C. ambrosioides* were effective against *T. granarium*, while that of *C. ambrosioides* caused 100% adult mortality of *T. castaneum* (Nenaah and Ibrahim, 2011). The LC_50_ value of *lambda-cyhalothrin* to quercetin-fed *H. armigera* larvae was 2.39-fold higher than the control. This indicates a reduced sensitivity to lambda-cyhalothrin^44^.

In wheat seeds a red flour beetles infestation were studied by Afsheen et al. (2019). Where cold plasma treatment is carried out at 800 V for 1 and 4 min. There the minimum grain weight loss is observed in plasma treated seeds as compared to the control. The minimum mortality rate occurred in the control seed and maximum at 4 min plasma treatment. Plasma treatment can be used against red flour beetles particularly for long-term storage by controlling the insecticidal effects^45^. The activity of essential oil from coriander essential oil against *Tribolium castaneum* studied by Islam et al.^46^. In their findings, *C. sativum* oil showed high repellent activity to the adults of *T. castaneum*, up to 90%. *C. sativum* oil used 12 µg/ml shown repellency to 100% in a filter paper arena test. Results in our investigation for all the parameters viz. grain damage percentage, grain weight loss percentage, number of eggs/grain, and number of holes/grain are similar with the results obtained by Afsheen et al. (2019) and Rahman et al.^34,45^.

In the present study, it can be stated that cold plasma processing is found significant to avoid pulse beetle growth, infestation, and feeding on the chickpea samples for about four years, which is a comparatively very long period after considering the nature of infestation and losses caused by pulse beetle.

## Materials and methods

### Materials

The four chickpea cultivars viz*. Kripa, Virat* (Kabuli*) Vishal, Rajas* (Desi) were procured from the Pulses Improvement Project, Mahatma Phule Krishi Vidyapeeth, Rahuri complying with relevant institutional, national, and international guidelines and legislation.

### Cold plasma device

**Figure 1:**
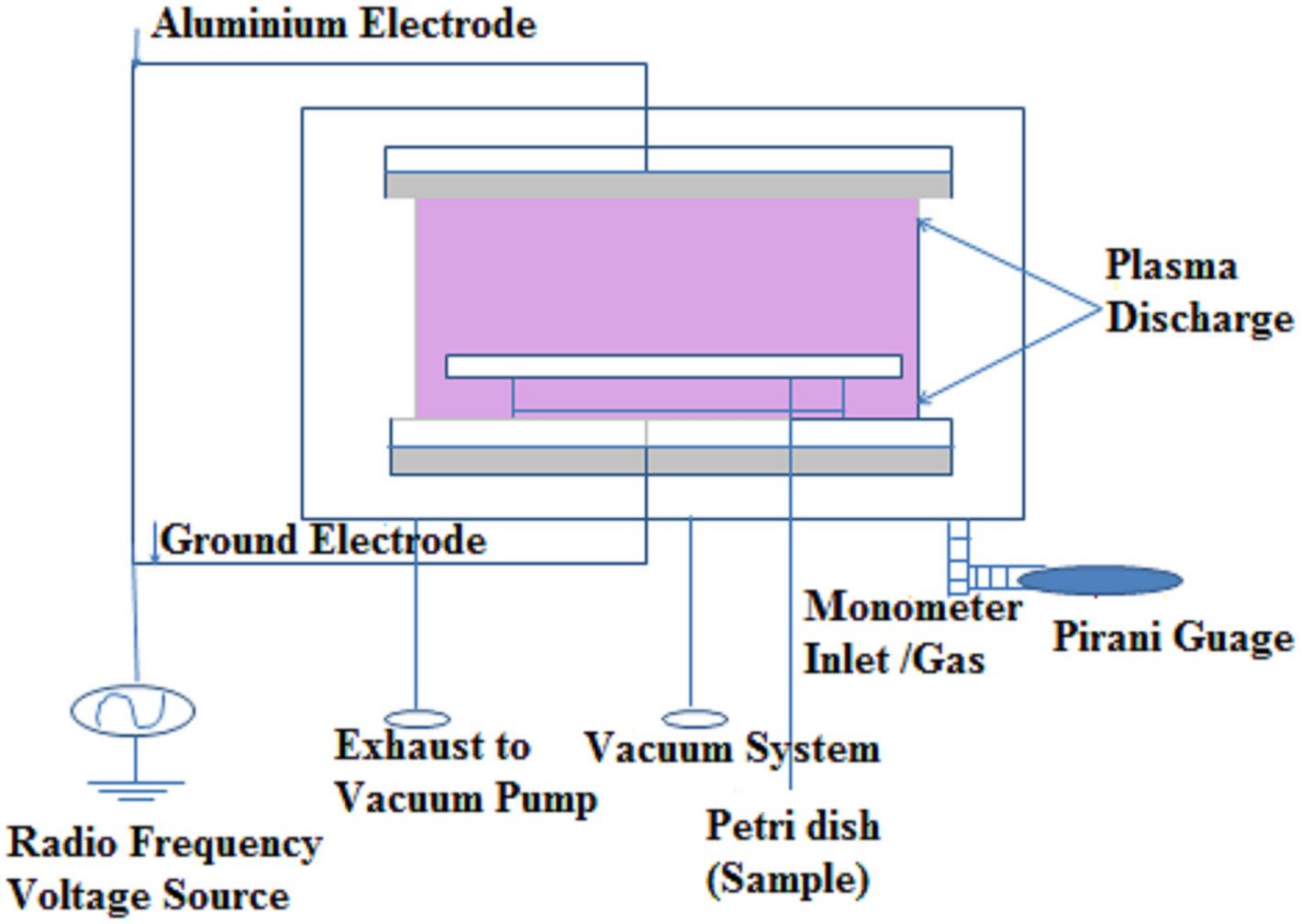
Schematic diagram of the low-temperature plasma system.

**Figure 2:**
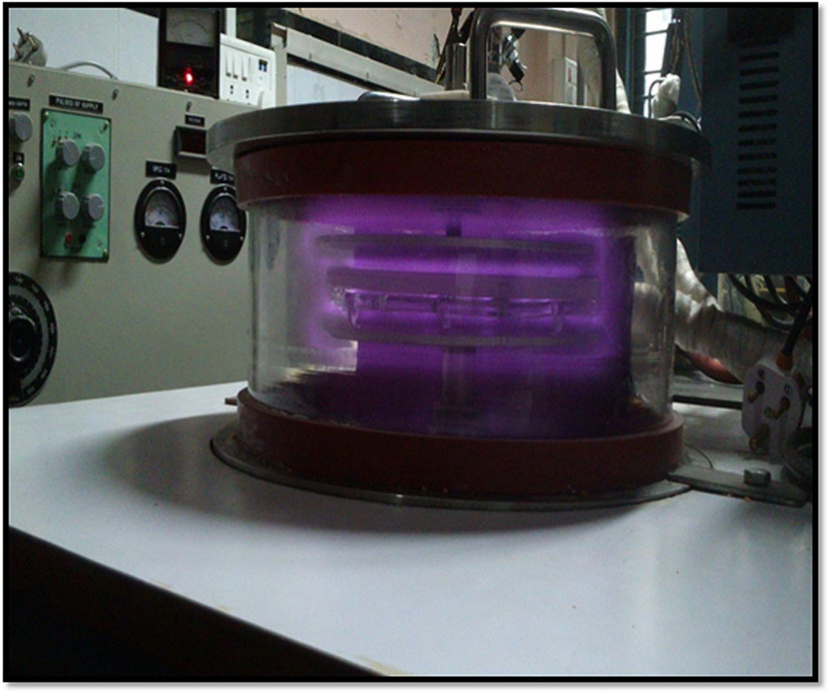
Photograph of the low-temperature plasma system.

An in-house designed low-pressure glow discharge plasma with bell-jar symmetry was employed for plasma treatment. The walls of the reactor are made up of Pyrex glass with 3 mm of thickness. The base and the opening lid are made up of stainless steel. The electrodes used were made of aluminum which has a 20 cm diameter. The electrode distance inside the reactor was maintained at 3 cm during all the plasma treatments where the chickpea seeds are exposed to capacitively coupled glow discharge plasma as shown in Figs. 1 and 2. The system is capacitively coupled with a radio frequency power source having a frequency of 13.56 MHz. The system pressure was initially achieved at 0.05 mBar with samples in the system by using HHV vacuum pump ED-20 and the working pressure was adjusted to the optimized value of 0.5 mBar. The plasma glow was observed (Figs. 1 and 2). The plasma treatment on chickpea cultivars was done at 40, 50, and 60 W each having an exposure time of 10, 15 and 20 min. Chickpea samples were kept spread in thin and less dense layer on the wire mesh to ensure uniform distribution of plasma across all the grains.

### Rearing of insects

Insect rearing was carried out under the prevailing environmental conditions of 30 ± 2 °C temperature and 70 ± 5 RH in the dark^35,36,37^. Newly emerged pulse beetles of same generation were obtained, by releasing 10 beetles viz. five pairs of newly emerged adult female bruchid insects in a plastic container with 100 g of chickpea seeds covered by a muslin cloth fastened with rubber band. All the adults were removed after 24 h and egg laid seeds were maintained at required temperature and humidity. The insects emerged after four weeks were used in the entire investigation. Insect eggs were counted by using hand lens. (Shukla 2007).

### Procedure for observation of pulse beetle infestation during storage

The four chickpea cultivars were exposed to 40, 50, and 60 W cold plasma treatment each having an exposure time of 10, 15, and 20 min. Plasma treated and untreated chickpeas 100 grains were stored in airtight zip lock pouches of each sample. Ziplock pouches were kept at room temperature (Average temperature 24 ± 4 °C, relative humidity 59 ± 5). During the storage period, the individual pouches were opened and checked critically for the presence of eggs, larvae, or adult pulse beetles and damage caused by them, at an interval of 3 months. The observations were noted and used for further calculations.

### Quarterly assessment of grain damage percentage

For determination of grain damage percentage.100-grains were taken from each zip lock pouch quarterly. The number of bored grains were separated out with the help of hand magnifying lens. The bored seeds were measured. The percent grain damage was calculated using the following Eq. ^51^:

1 $${\text{Chikpea}}\,{\text{grain}}\,{\text{damage}}\,{\text{percent}}\,{\text{(bored}}\,{\text{seed)}} = ({\text{Number}}\,{\text{of}}\,{\text{bored}}\,{\text{seeds}})/({\text{Total}}\,{\text{number}}\,{\text{of}}\,{\text{seeds}}\,{\text{of}}\,{\text{sample}}) \times 100$$

### Quarterly assessment of grain weight loss percentage

For the determination of grain weight loss percentage, 100-grains were taken from each zip lock pouch quarterly. Their weight is measured^47^. The percent weight loss was calculated using the following Eq. ^48^:

2 $${\text{Chickpea}}\,{\text{grain}}\,{\text{percent}}\,{\text{weight}}\,{\text{loss}} = ({\text{Initial}}\,{\text{Wt}}{\text{.}}\,{\text{of}}\,{\text{grains}} - {\text{Final}}\,{\text{Wt}}{\text{.}}\,{\text{of}}\,{\text{grains}})/({\text{Initial}}\,{\text{Wt}}{\text{.}}\,{\text{of}}\,{\text{grains}}) \times 100$$

### Quarterly assessment of the number of eggs/grain

For the determination of the number of eggs/grain, 100-grains were taken from each zip lock pouch quarterly. The presence of eggs of pulse beetles can be easily seen in infested pulses with the naked eye these are singly occurring yellow-colored, which became opaque when hatched^49,50^. The eggs on grains were measured. The number of eggs/grain was calculated.

### Quarterly assessment of the number of holes/grain

For determination of the number of holes/grain, 100-grains were taken from each zip lock pouch quarterly and grains with holes are counted. The number of holes/grain was calculated.

### Statistical analysis

The findings were statistically analyzed using SPSS (IBM statistical analysis version 19) using one-way ANOVA. The significance between the samples was compared at *p* < 0.05 where the least significant difference was tested by the Post-hoc and Duncan test. The averages from three different studies were presented in all of the findings.

### Ethical approval

This article does not contain any studies with human participants performed by any of the authors.

### Human and animal rights

The research did not involve human participants and/or animals.

## Conclusion

The cold plasma treatment has attained much importance in various areas of food processing. In the present era, scientists are finding practical alternatives to chemical fumigants. Based on all the previous studies discussed here like sex pheromones, genome editing technique, use of nanocarrier, sterile insect technique, nanoencapsulation of botanical compounds, use of bio fumigants, essential oils, use of thermal treatments, use of RNAi, cold plasma treatment found to be the best remedy for controlling the storage pests like pulse beetle or bruchid. Moreover, there are additional advantages of plasma processing to the chickpea seeds like its surface modification and subsequent high rate of moisture absorption, control of hard-to-cook phenomenon with improved soaking and cooking, improved germination, prevention of microbial or pathogen infection. It is providing prolonged storage to the pulses for few years which is otherwise difficult to store for few months. This research showed that cold plasma has an excellent resistive effect against *C. chinensis* and it can be used to control a wide range of storage pests to have sustainable storage pest control*.* Further studies are required to design and develop commercial and continuous cold plasma treatment machinery for the processing of chickpea grains on a commercial basis.

## Supplementary Information

Supplementary Information 1.

Supplementary Information 2.

Supplementary Information 3.

Supplementary Information 4.

Supplementary Information 5.

Supplementary Information 6.

Supplementary Information 7.

Supplementary Information 8.

## Data Availability

The authors declare that data supporting the findings of this study are available within the paper and its Supplementary Information files. Source data are provided with this paper.
