# Supplementary material for: Potential of cold plasma to control Callosobruchus chinensis (Chrysomelidae: Bruchinae) in chickpea cultivars during four year storage
Source: Sci Rep. 2021 Jun 28;11:13425. doi: 10.1038/s41598-021-92792-x (PMC8238940; doi:10.1038/s41598-021-92792-x)

Supplementary Figure S1. Quarterly Chickpea grain damage (%)

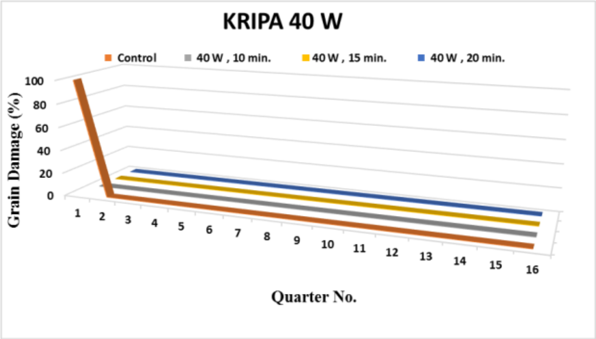

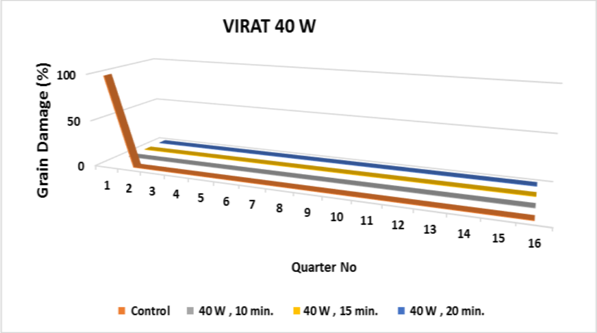

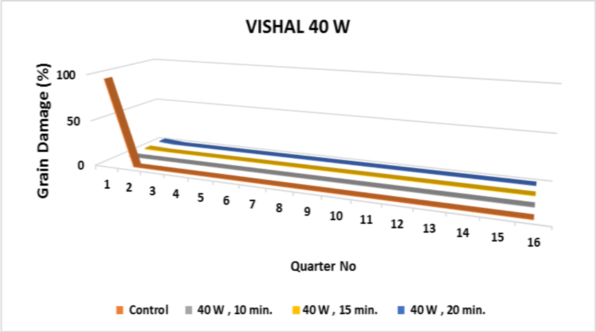

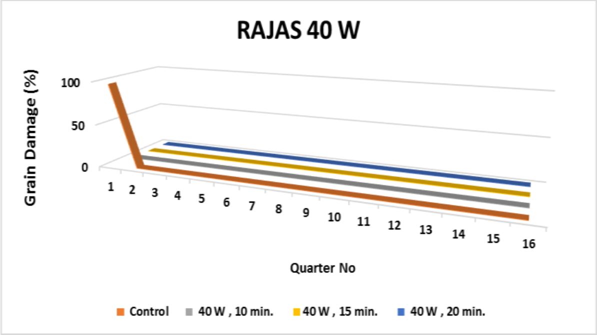

Supplementary Figure S1. Quarterly Chickpea grain damage (%)

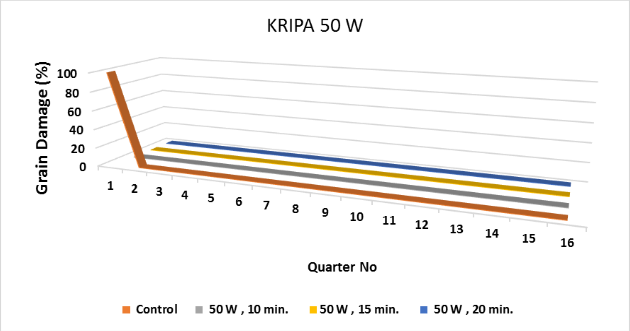

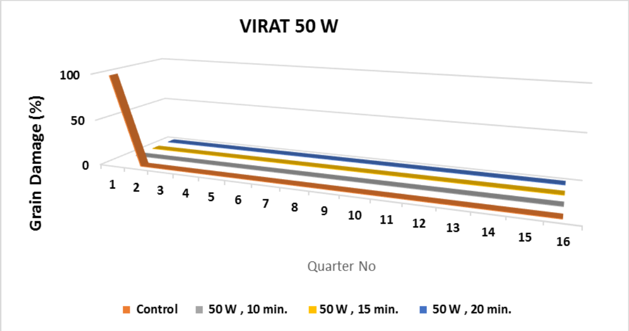

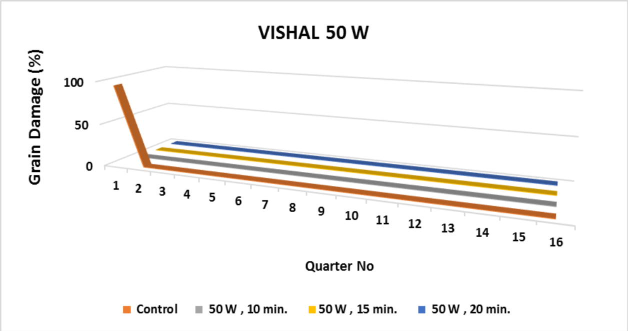

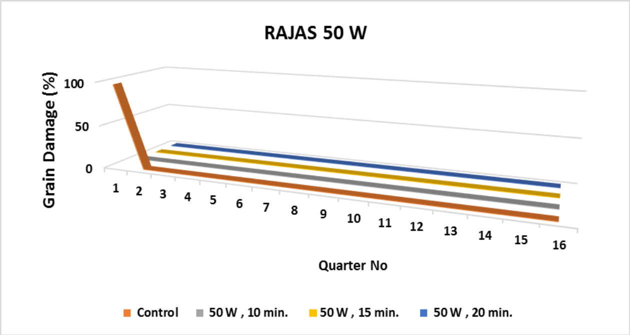

Supplementary Figure S1. Quarterly Chickpea grain damage (%)

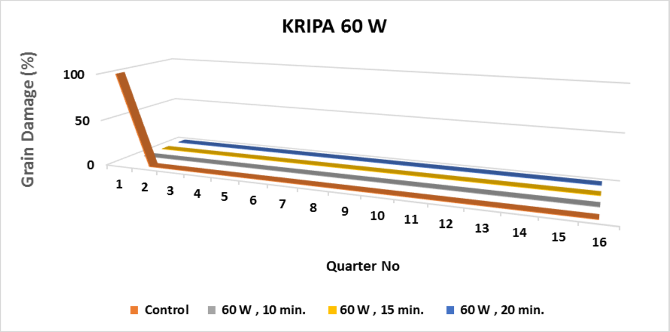

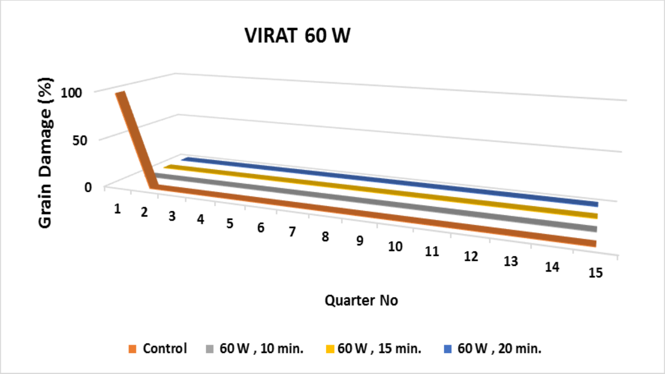

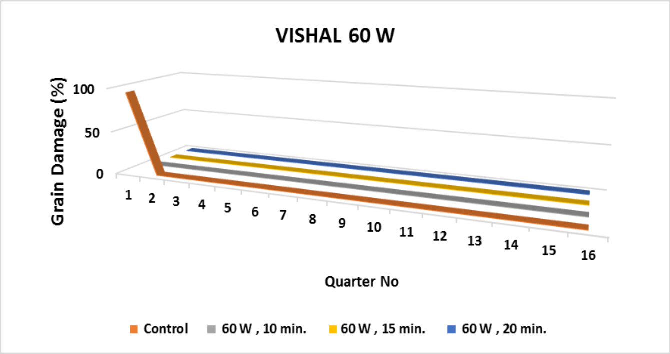

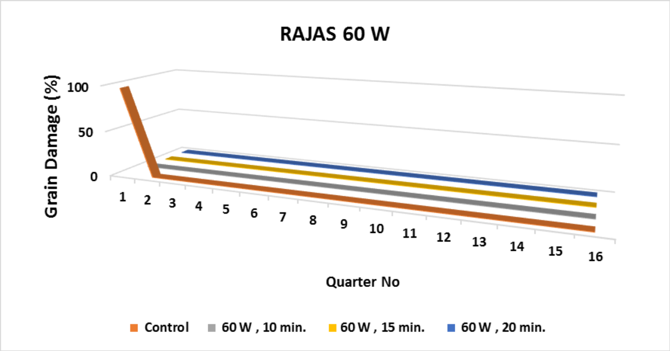

Supplement: Supplementary file 1 — Supplementary Information 1. [file 41598_2021_92792_MOESM1_ESM.pdf]
