# Supplementary material for: Potential of cold plasma to control Callosobruchus chinensis (Chrysomelidae: Bruchinae) in chickpea cultivars during four year storage
Source: Sci Rep. 2021 Jun 28;11:13425. doi: 10.1038/s41598-021-92792-x (PMC8238940; doi:10.1038/s41598-021-92792-x)
Supplement: Supplementary file 6 — Supplementary Information 6. [file 41598_2021_92792_MOESM6_ESM.pdf]

Supplementary Table S2 Quarterly Chickpea grain weight loss (%)

Kripa 40 W /Month	Control	40 W , 10 min.	40 W , 15 min.	40 W , 20 min.
3	56.87	0.25	0.27	0.26
6	57	0.5	0.53	0.51
9	57.44	0.75	0.76	0.73
12	58	1	1.02	1.03
15	58.33	1.23	1.25	1.24
18	59.07	1.48	1.49	1.47
21	59.83	1.64	1.68	1.68
24	60.3	2	2.1	2
27	60.8	4.24	4.26	4.27
30	61	4.45	4.48	4.49
33	61.34	4.71	4.73	4.75
36	61.76	4.91	4.93	4.96
39	61.99	5.3	5.32	5.33
42	62.34	5.33	5.34	5.39
45	62.79	5.7	5.73	5.99
48	62.99	6.4	6.43	6.55

Kripa 50 W/Month	Control	50 W , 10 min.	50 W , 15 min.	50 W , 20 min.
3	56.87	0.27	0.15	0.29
6	57	0.54	0.36	0.58
9	57.44	0.79	0.47	0.81
12	58	1.08	0.56	1.11
15	58.33	1.29	0.69	1.33
18	59.07	1.57	0.78	1.61
21	59.83	1.79	0.91	1.81
24	60.3	2.13	1.05	2.16
27	60.8	4.29	5.15	5.19
30	61	4.51	5.39	5.36
33	61.34	4.79	5.47	5.49
36	61.76	4.98	5.98	6.03
39	61.99	5.35	6.28	6.31
42	62.34	5.44	6.39	6.43
45	62.79	6.03	6.66	6.69
48	62.99	4.39	6.77	6.79

Kripa 60 W /Month	Control	60 W , 10 min.	60 W , 15 min.	60 W , 20 min.
3	56.87	0.16	0.26	0.15
6	57	0.37	0.51	0.35
9	57.44	0.49	0.76	0.47
12	58	0.59	1.01	0.57
15	58.33	0.71	1.25	0.69
18	59.07	0.81	1.49	0.78
21	59.83	0.94	1.66	0.92
24	60.3	1.09	2.01	1
27	60.8	5.22	5.25	5.29
30	61	5.39	5.44	5.48
33	61.34	5.53	5.57	5.59
36	61.76	6.11	6.15	6.18
39	61.99	6.35	6.39	6.42
42	62.34	6.45	6.49	6.53
45	62.79	6.49	6.53	6.56
48	62.99	6.54	6.57	6.59

Virat 40 W /Month	Control	40 W , 10 min.	40 W , 15 min.	40 W , 20 min.
3	58.14	1	1.04	1.02
6	58.44	1.78	1.84	1.86
9	58.86	2.31	2.41	2.44
12	59.11	2.82	2.92	2.95
15	59.49	3.51	3.59	3.56
18	59.87	3.86	3.99	3.98
21	60.12	4.37	4.47	4.46
24	60.42	4.81	4.9	4.92
27	60.86	5.31	5.4	5.42
30	61.01	5.81	5.91	5.96
33	61.34	6.3	6.43	6.44
36	61.79	6.86	6.97	6.98
39	61.97	7.5	7.57	7.59
42	62.07	8.61	8.69	8.68
45	62.44	8.85	9.14	9.04
48	62.66	9.03	9.65	10.52

Virat 50 W /Month	Control	50 W , 10 min.	50 W , 15 min.	50 W , 20 min.
3	58.14	1.01	1.11	1.03
6	58.44	1.81	1.98	1.86
9	58.86	2.39	2.49	2.44
12	59.11	2.88	2.94	2.97
15	59.49	3.55	3.59	3.59
18	59.87	3.9	3.98	3.98
21	60.12	4.42	4.47	4.46
24	60.42	4.85	4.95	4.93
27	60.86	5.36	5.48	5.43
30	61.01	5.87	5.98	5.98
33	61.34	6.39	6.49	6.45
36	61.79	6.93	7.17	6.99
39	61.97	7.54	7.87	7.59
42	62.07	8.65	8.89	8.72
45	62.44	9.04	9.34	9.36
48	62.66	9.26	9.92	10.55

Virat 60 W /Month	Control	60 W , 10 min.	60 W , 15 min.	60 W , 20 min.
3	58.14	1.01	1.01	1.02
6	58.44	1.81	1.84	2.06
9	58.86	2.38	2.42	3.04
12	59.11	2.87	2.94	4.05
15	59.49	3.56	3.55	5.06
18	59.87	3.96	3.96	6.18
21	60.12	4.45	4.44	7.06
24	60.42	4.85	4.9	8.222
27	60.86	5.37	5.41	9.02
30	61.01	5.89	5.95	9.66
33	61.34	6.39	6.42	10.04
36	61.79	6.93	6.97	10.78
39	61.97	7.52	7.56	11.09
42	62.07	8.63	8.69	11.68
45	62.44	9.24	9.33	12.04
48	62.66	9.49	10.49	12.34

Vishal 40 W /Month	Control	40 W , 10 min.	40 W , 15 min.	40 W , 20 min.
3	56.85	1	0.88	0.89
6	57.3	1.77	1.69	1.69
9	57.77	2.31	2.23	2.24
12	57.89	2.81	2.71	2.72
15	58.21	3.51	3.43	3.44
18	58.53	3.88	3.77	3.79
21	58.84	4.39	4.31	4.3
24	59.33	4.76	4.66	4.68
27	59.89	5.31	4.83	4.83
30	60.35	5.8	5.18	5.21
33	60.98	6.21	6	6.05
36	61.24	6.83	6.75	6.77
39	61.65	7.44	7.29	7.31
42	61.96	8.55	7.75	7.76
45	62.33	8.68	7.98	7.98
48	62.87	8.86	8.1	8.11

Vishal 50 W/Month	Control	50 W , 10 min.	50 W , 15 min.	50 W , 20 min.
3	56.85	0.85	0.77	0.83
6	57.3	1.64	1.56	1.62
9	57.77	2.21	2.15	2.18
12	57.89	2.7	2.64	2.66
15	58.21	3.41	3.34	3.39
18	58.53	3.75	3.69	3.71
21	58.84	4.27	4.19	4.21
24	59.33	4.65	4.54	4.61
27	59.89	4.81	4.77	4.79
30	60.35	5.19	4.99	5.15
33	60.98	6.01	5	6
36	61.24	6.73	5.8	6.75
39	61.65	7.28	6.11	7.21
42	61.96	7.75	6.54	7.69
45	62.33	7.93	6.87	7.88
48	62.87	8.04	7.15	8

Vishal 60 W /Month	Control	60 W , 10 min.	60 W , 15 min.	60 W , 20 min.
3	56.85	0.72	0.71	0.73
6	57.3	1.51	1.52	1.54
9	57.77	2.11	2.12	2.14
12	57.89	2.6	2.61	2.59
15	58.21	3.3	3.31	3.29
18	58.53	3.63	3.61	3.63
21	58.84	4.15	4.14	4.16
24	59.33	4.5	4.48	4.49
27	59.89	4.75	4.77	4.79
30	60.35	5.13	5.17	5.19
33	60.98	5.98	5.76	5.79
36	61.24	6.7	5.93	5.95
39	61.65	7.24	6.24	6.26
42	61.96	7.71	6.71	6.73
45	62.33	7.9	7.35	7.37
48	62.87	8.02	7.54	7.56

Rajas 40 W /Month	Control	40 W , 10 min.	40 W , 15 min.	40 W , 20 min.
3	56.83	1	1.25	0.89
6	57.03	2.09	2.99	1.89
9	57.56	3.33	3.59	2.42
12	57.93	4.03	4.99	3.88
15	58.34	5.06	5.94	4.76
18	58.79	6.09	6.99	5.93
21	59.03	7.04	7.74	6.49
24	59.53	8	8.36	7.87
27	59.89	9	9	9
30	60.35	9.49	9.88	9.33
33	60.98	9.88	10.38	10
36	61.24	10.35	10.75	10.88
39	61.65	10.55	11.15	11.23
42	61.96	10.96	11.86	11.63
45	62.33	11.12	12.24	11.68
48	62.87	11.3	12.46	12.3

Rajas 50 W /Month	Control	50 W , 10 min.	50 W , 15 min.	50 W , 20 min.
3	56.83	1.31	1.25	1.11
6	57.03	2.98	2.99	2.91
9	57.56	3.74	3.59	3.42
12	57.93	4.99	4.99	4.88
15	58.34	5.98	5.94	5.79
18	58.79	6.99	6.99	6.93
21	59.03	7.84	7.74	7.49
24	59.53	8.47	8.36	8.89
27	59.89	9.33	9.07	9.23
30	60.35	9.79	9.88	9.64
33	60.98	10.35	10.38	10.09
36	61.24	10.99	10.75	10.81
39	61.65	11.69	11.18	11.33
42	61.96	12.38	11.86	11.93
45	62.33	13.25	12.24	12.6
48	62.87	13.71	12.49	13.13

Rajas 60 W /Month	Control	60 W , 10 min.	60 W , 15 min.	60 W , 20 min.
3	56.83	1.47	1.49	1.25
6	57.03	2.97	2.95	2.95
9	57.56	3.69	3.69	3.66
12	57.93	4.99	4.9	4.99
15	58.34	5.99	5.99	5.94
18	58.79	6.99	6.91	6.99
21	59.03	7.98	7.98	7.74
24	59.53	8.99	8.94	8.36
27	59.89	9.43	9.53	9.25
30	60.35	9.83	9.89	9.73
33	60.98	10.48	10.78	10.27
36	61.24	10.99	10.99	10.94
39	61.65	11.88	11.98	11.56
42	61.96	12.99	13.09	12.28
45	62.33	13.98	14.78	12.96
48	62.87	14.83	15.01	13.46
