# Supplementary material for: Potential of cold plasma to control Callosobruchus chinensis (Chrysomelidae: Bruchinae) in chickpea cultivars during four year storage
Source: Sci Rep. 2021 Jun 28;11:13425. doi: 10.1038/s41598-021-92792-x (PMC8238940; doi:10.1038/s41598-021-92792-x)
Supplement: Supplementary file 7 — Supplementary Information 7. [file 41598_2021_92792_MOESM7_ESM.pdf]

**Supplementary Table 3. Quarterly number of eggs / grain**

| Kripa 40 W/ Month | Control | 40 W , 10 min. | 40 W , 15 min. | 40 W , 20 min. |
|---|---|---|---|---|
| 3 | 25.26 | 0 | 0 | 0 |
| 6 | 0 | 0 | 0 | 0 |
| 9 | 0 | 0 | 0 | 0 |
| 12 | 0 | 0 | 0 | 0 |
| 15 | 0 | 0 | 0 | 0 |
| 18 | 0 | 0 | 0 | 0 |
| 21 | 0 | 0 | 0 | 0 |
| 24 | 0 | 0 | 0 | 0 |
| 27 | 0 | 0 | 0 | 0 |
| 30 | 0 | 0 | 0 | 0 |
| 33 | 0 | 0 | 0 | 0 |
| 36 | 0 | 0 | 0 | 0 |
| 39 | 0 | 0 | 0 | 0 |
| 42 | 0 | 0 | 0 | 0 |
| 45 | 0 | 0 | 0 | 0 |
| 48 | 0 | 0 | 0 | 0 |

| Kripa 50 W/ Month | Control | 50 W , 10 min. | 50 W , 15 min. | 50 W , 20 min. |
|---|---|---|---|---|
| 3 | 25.26 | 0 | 0 | 0 |
| 6 | 0 | 0 | 0 | 0 |
| 9 | 0 | 0 | 0 | 0 |
| 12 | 0 | 0 | 0 | 0 |
| 15 | 0 | 0 | 0 | 0 |
| 18 | 0 | 0 | 0 | 0 |
| 21 | 0 | 0 | 0 | 0 |
| 24 | 0 | 0 | 0 | 0 |
| 27 | 0 | 0 | 0 | 0 |
| 30 | 0 | 0 | 0 | 0 |
| 33 | 0 | 0 | 0 | 0 |
| 36 | 0 | 0 | 0 | 0 |
| 39 | 0 | 0 | 0 | 0 |
| 42 | 0 | 0 | 0 | 0 |
| 45 | 0 | 0 | 0 | 0 |
| 48 | 0 | 0 | 0 | 0 |

| Kripa 60 W / Month | Control | 60 W , 10 min. | 60 W , 15 min. | 60 W , 20 min. |
|---|---|---|---|---|
| 3 | 25.26 | 0 | 0 | 0 |
| 6 | 0 | 0 | 0 | 0 |
| 9 | 0 | 0 | 0 | 0 |
| 12 | 0 | 0 | 0 | 0 |
| 15 | 0 | 0 | 0 | 0 |
| 18 | 0 | 0 | 0 | 0 |
| 21 | 0 | 0 | 0 | 0 |
| 24 | 0 | 0 | 0 | 0 |
| 27 | 0 | 0 | 0 | 0 |
| 30 | 0 | 0 | 0 | 0 |
| 33 | 0 | 0 | 0 | 0 |
| 36 | 0 | 0 | 0 | 0 |
| 39 | 0 | 0 | 0 | 0 |
| 42 | 0 | 0 | 0 | 0 |
| 45 | 0 | 0 | 0 | 0 |
| 48 | 0 | 0 | 0 | 0 |

| Virat 40 W / Month | Control | 40 W , 10 min. | 40 W , 15 min. | 40 W , 20 min. |
|---|---|---|---|---|
| 3 | 24.23 | 0 | 0 | 0 |
| 6 | 0 | 0 | 0 | 0 |
| 9 | 0 | 0 | 0 | 0 |
| 12 | 0 | 0 | 0 | 0 |
| 15 | 0 | 0 | 0 | 0 |
| 18 | 0 | 0 | 0 | 0 |
| 21 | 0 | 0 | 0 | 0 |
| 24 | 0 | 0 | 0 | 0 |
| 27 | 0 | 0 | 0 | 0 |
| 30 | 0 | 0 | 0 | 0 |
| 33 | 0 | 0 | 0 | 0 |
| 36 | 0 | 0 | 0 | 0 |
| 39 | 0 | 0 | 0 | 0 |
| 42 | 0 | 0 | 0 | 0 |
| 45 | 0 | 0 | 0 | 0 |
| 48 | 0 | 0 | 0 | 0 |

| Virat 50 W / Month | Control | 50 W , 10 min. | 50 W , 15 min. | 50 W , 20 min. |
|---|---|---|---|---|
| 3 | 24.23 | 0 | 0 | 0 |
| 6 | 0 | 0 | 0 | 0 |
| 9 | 0 | 0 | 0 | 0 |
| 12 | 0 | 0 | 0 | 0 |
| 15 | 0 | 0 | 0 | 0 |
| 18 | 0 | 0 | 0 | 0 |
| 21 | 0 | 0 | 0 | 0 |
| 24 | 0 | 0 | 0 | 0 |
| 27 | 0 | 0 | 0 | 0 |
| 30 | 0 | 0 | 0 | 0 |
| 33 | 0 | 0 | 0 | 0 |
| 36 | 0 | 0 | 0 | 0 |
| 39 | 0 | 0 | 0 | 0 |
| 42 | 0 | 0 | 0 | 0 |
| 45 | 0 | 0 | 0 | 0 |
| 48 | 0 | 0 | 0 | 0 |

| Virat 60 W / Month | Control | 60 W , 10 min. | 60 W , 15 min. | 60 W , 20 min. |
|---|---|---|---|---|
| 3 | 24.23 | 0 | 0 | 0 |
| 6 | 0 | 0 | 0 | 0 |
| 9 | 0 | 0 | 0 | 0 |
| 12 | 0 | 0 | 0 | 0 |
| 15 | 0 | 0 | 0 | 0 |
| 18 | 0 | 0 | 0 | 0 |
| 21 | 0 | 0 | 0 | 0 |
| 24 | 0 | 0 | 0 | 0 |
| 27 | 0 | 0 | 0 | 0 |
| 30 | 0 | 0 | 0 | 0 |
| 33 | 0 | 0 | 0 | 0 |
| 36 | 0 | 0 | 0 | 0 |
| 39 | 0 | 0 | 0 | 0 |
| 42 | 0 | 0 | 0 | 0 |
| 45 | 0 | 0 | 0 | 0 |
| 48 | 0 | 0 | 0 | 0 |

| Vishal 40 W / Month | Control | 40 W , 10 min. | 40 W , 15 min. | 40 W , 20 min. |
|---|---|---|---|---|
| 3 | 24.6 | 0 | 1 | 2 |
| 6 | 0 | 0 | 0 | 0 |
| 9 | 0 | 0 | 0 | 0 |
| 12 | 0 | 0 | 0 | 0 |
| 15 | 0 | 0 | 0 | 0 |
| 18 | 0 | 0 | 0 | 0 |
| 21 | 0 | 0 | 0 | 0 |
| 24 | 0 | 0 | 0 | 0 |
| 27 | 0 | 0 | 0 | 0 |
| 30 | 0 | 0 | 0 | 0 |
| 33 | 0 | 0 | 0 | 0 |
| 36 | 0 | 0 | 0 | 0 |
| 39 | 0 | 0 | 0 | 0 |
| 42 | 0 | 0 | 0 | 0 |
| 45 | 0 | 0 | 0 | 0 |
| 48 | 0 | 0 | 0 | 0 |

| Vishal 50 W / Month | Control | 50 W , 10 min. | 50 W , 15 min. | 50 W , 20 min. |
|---|---|---|---|---|
| 3 | 24.6 | 0 | 0 | 0 |
| 6 | 0 | 0 | 0 | 0 |
| 9 | 0 | 0 | 0 | 0 |
| 12 | 0 | 0 | 0 | 0 |
| 15 | 0 | 0 | 0 | 0 |
| 18 | 0 | 0 | 0 | 0 |
| 21 | 0 | 0 | 0 | 0 |
| 24 | 0 | 0 | 0 | 0 |
| 27 | 0 | 0 | 0 | 0 |
| 30 | 0 | 0 | 0 | 0 |
| 33 | 0 | 0 | 0 | 0 |
| 36 | 0 | 0 | 0 | 0 |
| 39 | 0 | 0 | 0 | 0 |
| 42 | 0 | 0 | 0 | 0 |
| 45 | 0 | 0 | 0 | 0 |
| 48 | 0 | 0 | 0 | 0 |

| Vishal 60 W / Month | Control | 60 W , 10 min. | 60 W , 15 min. | 60 W , 20 min. |
|---|---|---|---|---|
| 3 | 24.6 | 0 | 0 | 0 |
| 6 | 0 | 0 | 0 | 0 |
| 9 | 0 | 0 | 0 | 0 |
| 12 | 0 | 0 | 0 | 0 |
| 15 | 0 | 0 | 0 | 0 |
| 18 | 0 | 0 | 0 | 0 |
| 21 | 0 | 0 | 0 | 0 |
| 24 | 0 | 0 | 0 | 0 |
| 27 | 0 | 0 | 0 | 0 |
| 30 | 0 | 0 | 0 | 0 |
| 33 | 0 | 0 | 0 | 0 |
| 36 | 0 | 0 | 0 | 0 |
| 39 | 0 | 0 | 0 | 0 |
| 42 | 0 | 0 | 0 | 0 |
| 45 | 0 | 0 | 0 | 0 |
| 48 | 0 | 0 | 0 | 0 |

| Rajas 40 W / Month | Control | 40 W , 10 min. | 40 W , 15 min. | 40 W , 20 min. |
|---|---|---|---|---|
| 3 | 25.44 | 0 | 0 | 0 |
| 6 | 0 | 0 | 0 | 0 |
| 9 | 0 | 0 | 0 | 0 |
| 12 | 0 | 0 | 0 | 0 |
| 15 | 0 | 0 | 0 | 0 |
| 18 | 0 | 0 | 0 | 0 |
| 21 | 0 | 0 | 0 | 0 |
| 24 | 0 | 0 | 0 | 0 |
| 27 | 0 | 0 | 0 | 0 |
| 30 | 0 | 0 | 0 | 0 |
| 33 | 0 | 0 | 0 | 0 |
| 36 | 0 | 0 | 0 | 0 |
| 39 | 0 | 0 | 0 | 0 |
| 42 | 0 | 0 | 0 | 0 |
| 45 | 0 | 0 | 0 | 0 |
| 48 | 0 | 0 | 0 | 0 |

| Rajas 50 W / Month | Control | 50 W , 10 min. | 50 W , 15 min. | 50 W , 20 min. |
|---|---|---|---|---|
| 3 | 25.44 | 0 | 0 | 0 |
| 6 | 0 | 0 | 0 | 0 |
| 9 | 0 | 0 | 0 | 0 |
| 12 | 0 | 0 | 0 | 0 |
| 15 | 0 | 0 | 0 | 0 |
| 18 | 0 | 0 | 0 | 0 |
| 21 | 0 | 0 | 0 | 0 |
| 24 | 0 | 0 | 0 | 0 |
| 27 | 0 | 0 | 0 | 0 |
| 30 | 0 | 0 | 0 | 0 |
| 33 | 0 | 0 | 0 | 0 |
| 36 | 0 | 0 | 0 | 0 |
| 39 | 0 | 0 | 0 | 0 |
| 42 | 0 | 0 | 0 | 0 |
| 45 | 0 | 0 | 0 | 0 |
| 48 | 0 | 0 | 0 | 0 |

| Rajas 60 W / Month | Control | 60 W , 10 min. | 60 W , 15 min. | 60 W , 20 min. |
|---|---|---|---|---|
| 3 | 25.44 | 0 | 0 | 0 |
| 6 | 0 | 0 | 0 | 0 |
| 9 | 0 | 0 | 0 | 0 |
| 12 | 0 | 0 | 0 | 0 |
| 15 | 0 | 0 | 0 | 0 |
| 18 | 0 | 0 | 0 | 0 |
| 21 | 0 | 0 | 0 | 0 |
| 24 | 0 | 0 | 0 | 0 |
| 27 | 0 | 0 | 0 | 0 |
| 30 | 0 | 0 | 0 | 0 |
| 33 | 0 | 0 | 0 | 0 |
| 36 | 0 | 0 | 0 | 0 |
| 39 | 0 | 0 | 0 | 0 |
| 42 | 0 | 0 | 0 | 0 |
| 45 | 0 | 0 | 0 | 0 |
| 48 | 0 | 0 | 0 | 0 |
