# Supplementary material for: Potential of cold plasma to control Callosobruchus chinensis (Chrysomelidae: Bruchinae) in chickpea cultivars during four year storage
Source: Sci Rep. 2021 Jun 28;11:13425. doi: 10.1038/s41598-021-92792-x (PMC8238940; doi:10.1038/s41598-021-92792-x)
Supplement: Supplementary file 8 — Supplementary Information 8. [file 41598_2021_92792_MOESM8_ESM.pdf]

Supplementary Table S4. Quarterly number of holes/ grain

Kripa 40 W /Month	Control	40 W , 10 min.	40 W , 15 min.	40 W , 20 min.
3	3.51	0	0	0
6	0	0	0	0
9	0	0	0	0
12	0	0	0	0
15	0	0	0	0
18	0	0	0	0
21	0	0	0	0
24	0	0	0	0
27	0	0	0	0
30	0	0	0	0
33	0	0	0	0
36	0	0	0	0
39	0	0	0	0
42	0	0	0	0
45	0	0	0	0
48	0	0	0	0

Kripa 50 W /Month	Control	50 W , 10 min.	50 W , 15 min.	50 W , 20 min.
3	3.51	0	0	0
6	0	0	0	0
9	0	0	0	0
12	0	0	0	0
15	0	0	0	0
18	0	0	0	0
21	0	0	0	0
24	0	0	0	0
27	0	0	0	0
30	0	0	0	0
33	0	0	0	0
36	0	0	0	0
39	0	0	0	0
42	0	0	0	0
45	0	0	0	0
48	0	0	0	0

Kripa 60 W /Month	Control	60 W , 10 min.	60 W , 15 min.	60 W , 20 min.
3	3.51	0	0	0
6	0	0	0	0
9	0	0	0	0
12	0	0	0	0
15	0	0	0	0
18	0	0	0	0
21	0	0	0	0
24	0	0	0	0
27	0	0	0	0
30	0	0	0	0
33	0	0	0	0
36	0	0	0	0
39	0	0	0	0
42	0	0	0	0
45	0	0	0	0
48	0	0	0	0

Virat 40 W /Month	Control	40 W , 10 min.	40 W , 15 min.	40 W , 20 min.
3	3.14	0	0	0
6	0	0	0	0
9	0	0	0	0
12	0	0	0	0
15	0	0	0	0
18	0	0	0	0
21	0	0	0	0
24	0	0	0	0
27	0	0	0	0
30	0	0	0	0
33	0	0	0	0
36	0	0	0	0
39	0	0	0	0
42	0	0	0	0
45	0	0	0	0
48	0	0	0	0

Virat 50 W /Month	Control	50 W , 10 min.	50 W , 15 min.	50 W , 20 min.
3	3.14	0	0	0
6	0	0	0	0
9	0	0	0	0
12	0	0	0	0
15	0	0	0	0
18	0	0	0	0
21	0	0	0	0
24	0	0	0	0
27	0	0	0	0
30	0	0	0	0
33	0	0	0	0
36	0	0	0	0
39	0	0	0	0
42	0	0	0	0
45	0	0	0	0
48	0	0	0	0

Virat 60 W /Month	Control	60 W , 10 min.	60 W , 15 min.	60 W , 20 min.
3	3.14	0	0	0
6	0	0	0	0
9	0	0	0	0
12	0	0	0	0
15	0	0	0	0
18	0	0	0	0
21	0	0	0	0
24	0	0	0	0
27	0	0	0	0
30	0	0	0	0
33	0	0	0	0
36	0	0	0	0
39	0	0	0	0
42	0	0	0	0
45	0	0	0	0
48	0	0	0	0

Vishal 40 W /Montl	Control	40 W , 10 min.	40 W , 15 min.	40 W , 20 min.
3	2.9	0	0.01	0.02
6	0	0	0	0
9	0	0	0	0
12	0	0	0	0
15	0	0	0	0
18	0	0	0	0
21	0	0	0	0
24	0	0	0	0
27	0	0	0	0
30	0	0	0	0
33	0	0	0	0
36	0	0	0	0
39	0	0	0	0
42	0	0	0	0
45	0	0	0	0
48	0	0	0	0

Vishal 50 W /Montl	Control	50 W , 10 min.	50 W , 15 min.	50 W , 20 min.
3	2.9	0	0	0
6	0	0	0	0
9	0	0	0	0
12	0	0	0	0
15	0	0	0	0
18	0	0	0	0
21	0	0	0	0
24	0	0	0	0
27	0	0	0	0
30	0	0	0	0
33	0	0	0	0
36	0	0	0	0
39	0	0	0	0
42	0	0	0	0
45	0	0	0	0
48	0	0	0	0

Vishal 60 W /Montl	Control	60 W , 10 min.	60 W , 15 min.	60 W , 20 min.
3	2.9	0	0	0
6	0	0	0	0
9	0	0	0	0
12	0	0	0	0
15	0	0	0	0
18	0	0	0	0
21	0	0	0	0
24	0	0	0	0
27	0	0	0	0
30	0	0	0	0
33	0	0	0	0
36	0	0	0	0
39	0	0	0	0
42	0	0	0	0
45	0	0	0	0
48	0	0	0	0

Rajas 40 W /Month	Control	40 W , 10 min.	40 W , 15 min.	40 W , 20 min.
3	3.12	0	0	0
6	0	0	0	0
9	0	0	0	0
12	0	0	0	0
15	0	0	0	0
18	0	0	0	0
21	0	0	0	0
24	0	0	0	0
27	0	0	0	0
30	0	0	0	0
33	0	0	0	0
36	0	0	0	0
39	0	0	0	0
42	0	0	0	0
45	0	0	0	0
48	0	0	0	0

Rajas 50 W /Month	Control	50 W , 10 min.	50 W , 15 min.	50 W , 20 min.
3	3.12	0	0	0
6	0	0	0	0
9	0	0	0	0
12	0	0	0	0
15	0	0	0	0
18	0	0	0	0
21	0	0	0	0
24	0	0	0	0
27	0	0	0	0
30	0	0	0	0
33	0	0	0	0
36	0	0	0	0
39	0	0	0	0
42	0	0	0	0
45	0	0	0	0
48	0	0	0	0

Rajas 60 W /Month	Control	60 W , 10 min.	60 W , 15 min.	60 W , 20 min.
3	3.12	0	0	0
6	0	0	0	0
9	0	0	0	0
12	0	0	0	0
15	0	0	0	0
18	0	0	0	0
21	0	0	0	0
24	0	0	0	0
27	0	0	0	0
30	0	0	0	0
33	0	0	0	0
36	0	0	0	0
39	0	0	0	0
42	0	0	0	0
45	0	0	0	0
48	0	0	0	0
